# Supplementary material for: Dynamic livelihood impacts of COVID-19 on different rural households in mountainous areas of China
Source: PLoS One. 2022 Sep 23;17(9):e0273816. doi: 10.1371/journal.pone.0273816 (PMC9506612; doi:10.1371/journal.pone.0273816)
Supplement: Attachment — Submitted filename: 0724Response to editors and reviewers.doc [file pone.0273816.s002.doc]

**Response to editors and reviewers**

**Dear** **editors and reviewers:**

Thank you very much for your careful consideration and constructive suggestions with regard to our manuscript: Dynamic Livelihood Impacts of COVID-19 on Different Rural Households in Mountainous Areas of China.

The comments are helpful for us to revise and improve the manuscript. The manuscript has now been revised according to these comments, using "Track Changes".

We appreciate Editors/Reviewers’ warm work earnestly, and expect that the revision will address your concerns about this manuscript. Please kindly contact us with any further questions.

**Responses to Editors’ comments:**

**Comment 1**:

1. Thank you for stating the following financial disclosure:

"The funders had no role in study design, data collection and analysis, decision to publish, or preparation of the manuscript."

At this time, please address the following queries:

a) Please clarify the sources of funding (financial or material support) for your study. List the grants or organizations that supported your study, including funding received from your institution.

b) State what role the funders took in the study. If the funders had no role in your study, please state: “The funders had no role in study design, data collection and analysis, decision to publish, or preparation of the manuscript.”

c) If any authors received a salary from any of your funders, please state which authors and which funders.

d)If you did not receive any funding for this study, please state: “The authors received no specific funding for this work.”

Please include your amended statements within your cover letter; we will change the online submission form on your behalf.

**Response**:

This research was jointly funded by the National Natural Science Foundation of China (Grant No. 4217011056) and Humanity and Social Science Youth Foundation of Chinese Ministry of Education (Grant No. 20YJCZH112). The funders had no role in study design, data collection and analysis, decision to publish, or preparation of the manuscript. The authors received no specific funding for this work

**Comment 2**:

2. We note that your Data Availability statement states the following: "All relevant data are within the manuscript and its Supporting Information files."

However, no Supporting Information files have been included in your submission

**Response**:

We supplemented the data in Supporting Information files

**Comment 3**:

Before we can proceed, please clarify the following:

1. Are there legal or ethical restrictions being placed upon the data? If so, please explain them in detail (e.g., data contain potentially identifying or sensitive patient information, data are owned by a third-party organization, etc.) and who has imposed them (e.g., a Research Ethics Committee or Institutional Review Board, etc.).

**Response**:

No legal or ethical restrictions are placed upon the data.

2. If the data contain identifying information, can the data be anonymized and be made publicly available?

**Response**:

Yes, it can be anonymized and be made publicly available.

3. Please note that PLOS does not allow authors to be the sole contact for data inquiries. If the data is only available upon request, please provide contact information, such as an email address, for a non-author, institutional point of contact (such as an IRB or ethics committee contact) who can field data inquiries from fellow researchers. If the data contact is an individual, please provide their title and relationship to the data as well.

**Response**:

We revised our MS according to the comment.

**Reviewer #1:**

**Comment 1**:

The manuscript is technically solid, the data obtained by the authors supports the investigation and its conclusions, and thus the study exhibits novel research findings: i find it especially important to focus on those households, those individuals, who were not in the center of attention of different government policies, especially the vulnerable groups we should talk about, in order to understand the real consequences of the global pandemic and the lockdowns.

According to the given data, the authors have not previously published this study. The paper's format is logical, i would say a bit short, but the main elements were included. The studies are carried out to a high technical degree and are sufficiently detailed within the publication, demonstrating that the analysis was carried out appropriately and systematically. The authors have included all data supporting their findings in their publication in tables, along with explanations for those tables. Additionally, the conclusion is given in the appropriate manner, the evidence supports the conclusions. The research complies with all applicable ethical and research integrity standards, and the work adheres to appropriate reporting criteria and community standards for data access.

**Response**:

We are grateful for your constructive suggestion and positive appraisal on our manuscript, we will further improve it to according to the comments.

**Reviewer #2:**

**Comment 1**:

The sample size is a severe problem. There are three sub problems:

1a: The study is supposed to look at impacts on rural China. Unfortunately, rural China has 700,000 villages. This study went to 2 of them. This needs to be addressed. It might be a fatal flaw (we really learn nothing about anything more than 2 villages).

**Response**:

Thank you for your valuable remind and constructive advice. The representativeness of the samples is the fundamental to survey research. Appropriate sampling methods and a certain scale of sample size is necessary for a national study.

We randomly selected these two villages, which does not mean that they represent 700,000 villages in China, but only represent common villages which consider fruit farming as the leading industry in mountainous agricultural county in southern China. Through in-depth investigation, some profound viewpoints could be discovered. Some missing information may produce some misunderstanding. We supplemented some important information in “*2.1 Study Area*” section according to the comment. Please see our revised MS.

**Comment 1**:

1b: The authors do not put their village in the context of other villages. This at least needs to be done.

**Response**:

We revised our MS according to the comment.

**Comment 1**:

1c: The authors only survey 95 households and then divide them into 4 groups. This means on average there are less than 25 sample observations per group. I again dear this undermines the convincing nature of the study.

**Response**:

Thanks for your important question. Some important explanation was neglected in our MS. We supplemented some information in our revised MS as follows: “It should be noted that there are many factors influencing our sample size: travel limitations and high risk of infection, high homogeneity and financial limitations. First, strict disease-control measures (including restrictions of trans-regional travels, reporting travel plan to governments) have been still adopted during our survey period in order to prevent the spread of COVID-19. It is not easy to gain the travel permission if you have not social networks there. And the potential risk of infection was higher because our investigators need to survey many interviewees who may just returned from medium-high risk areas. Second, our pilot survey indicated that there was high homogeneity in livelihood impacts among the same type of rural households. Third, the survey cost per respondent was very high owing to additional costs. However, the small sample size basically could not affect the research results because of qualitative research and high quality of survey data.”

In addition, our study was a case study which indicated the livelihood impacts of a mountainous agricultural county with developed fruit farming in southern China. Special agriculture in southern China is very developed in some mountains. The livelihood impacts of COVID-19 were different from these of rural areas mainly planting grain crops. The method of qualitative research has been adopted, which need less surveyed samples.

**Major Comment 2**:

The authors only asked 21 questions. In a standard survey of farm households it takes an entire section of questions to get a good estimate of income. Not to mention the other issues (eg, impact on child’s education; impact on consumer prices; etc). Plus, the authors were comparing to the year before. I have high doubts about the ability of such a data set to cast clear light on the issues at hand.

**Response**:

We are appreciated that you identified our errors. “21” should be replaced by “57”. Please see our revised MS.

**Major Comment 3**:

In the discussion section, the authors compare their results to other countries. This is good. However, we need to know more about the papers and comparisons. More discussion is needed.

**Response**:

Thanks for the comment. We added more discussion to in our revised MS.

**Major Comment 4:**

There are many papers on rural China that are not cited. For example, there are three papers by Dr. Huan Wang and her team from Stanford that use data from 700 villages and 7 provinces that are published in SCI and SSCI journals that are not cited. This illustrates a problem in their literature review. Have they missed other papers?

**Response**:

We revised our MS according to the comment.

**Major Comment 5**:

There is no set of limitations and strengths in the paper. These are needed.

**Response**:

We revised our MS according to the comment.

Reviewer #3:

**Major Comment 1**:

Data on victims of the pandemic need to be updated at line 26-29, Page 1.

**Response**:

Thanks for the comment. We have updated data on pandemic victims.

**Major Comment 2**:

Introduction: add some recent studies, especially related studies of China.

**Response**:

We appreciate your constructive comments. We supplemented included some recent literature in the manuscript according to the comment.

**Major Comment 3**:

Reasons to select the study case need to be highlighted.

**Response**:

We revised our MS according to the comment.

**Major Comment 4**:

Discussion: the spatial difference of the livelihood impacts should be emphasized. Especially, the Shanghai City has experienced 2 months lockdown. If the livelihood impacts and responses of rural households around the city were similar to these of the research.

**Response**:

We revised our MS according to the comment.

**Major Comment 5**:

Policy implication of the study should be supplemented in the last section.

**Response**:

We revised our MS according to the comment.
